# Perceptions of Aging from Persons Living and Aging with HIV: A Qualitative Study

**DOI:** 10.3390/healthcare14131879

**Published:** 2026-06-27

**Authors:** Shelby Brage, Manuel Ramos, Bruce Hirsch, Joseph McGowan, Christian Nouryan, Steven Y. Hong, Edith Burns

**Affiliations:** 1 Institute of Health Systems Science, Feinstein Institutes for Medical Research, Manhasset, NY 11030, USA; sbrage@northwell.edu (S.B.); jmcgowan@northwell.edu (J.M.); cnouryan@northwell.edu (C.N.); shong12@northwell.edu (S.Y.H.); 2 Zucker School of Medicine at Hofstra/Northwell, Hempstead, NY 11549, USA; mramos14@pride.hofstra.edu (M.R.); bhirsch@northwell.edu (B.H.); 3 Center for AIDS Research and Treatment, Northwell Health, Manhasset, NY 11030, USA

**Keywords:** HIV/AIDS, aging, qualitative methods, thematic analysis, social determinants of health

## Abstract

**Highlights:**

**What are the main findings?**

- PAWHs experience HIV independently from other chronic health conditions common with aging.
- A significant proportion express concern for growing isolation and future cognitive impairment.

**What are the implications of the main findings?**

- Clinicians may benefit from education on mental models of aging in the context of chronic conditions.
- Patients should be asked about social support and anticipate that those experiencing lower levels might need additional resources in the future.

**Abstract:**

**Background/Objectives:** People aging with HIV (PAWHs) face distinct health challenges, including early onset of aging and heightened risk for chronic comorbidities despite effective antiretroviral therapy (ART). However, significant gaps persist in understanding the lived experience and how PAWHs perceive the interplay between their controlled HIV and the aging process. This study examined PAWHs’ illness perceptions of aging, health, and relationship of HIV to other health conditions. **Methods:** Semi-structured interviews were conducted with a convenience sample of 25 PAWHs (mean age 63.5; mean time living with HIV 22.3 years; 24 virally suppressed) recruited through an academic HIV specialty clinic. Demographic and clinical data were collected from Electronic Health Records (EHRs), and interviews were analyzed using inductive thematic analysis. **Results:** A central finding was the disconnect between participants’ illness perceptions of controlled HIV and other aging-related health concerns. Absence of acute somatic symptoms and sustained viral suppression fostered a view of HIV as chronologically remote, leading to an apparent unawareness of HIV’s systemic links to accelerated aging and comorbidities. Two primary themes around aging emerged: acceptance/disengagement and fear of future debility (prevalent among older, socially isolated individuals concerned about dementia and finances). **Conclusions:** This pervasive disconnect, understandable through the lens of the Common Sense Model of Self-Regulation, highlights a critical need to adjust health counseling strategies for PAWHs. Clinicians can leverage existing trusted provider relationships to explicitly address and refine PAWHs’ illness models, clarifying that viral suppression is not a cure and educating on HIV’s systemic links to chronic conditions (e.g., ‘inflammaging’). Tailored educational interventions are crucial for fostering shared decision-making, encouraging early screening, and improving health outcomes for this vulnerable and growing population. Generalizability may be limited by sample characteristics.

## 1. Introduction

In the US, there are over half a million persons aging with HIV (PAWHs), defined as those aged 50 years and older [1], comprising more than 50% of all persons living with HIV (PLWHs). Projections estimate that by 2030, more than 70% of PLWHs will be aged ≥50 [2]. Improvements in anti-retrovirus treatment (ART) over the past years have contributed to these changing demographics, resulting in PAWHs having a similar life expectancy to those without HIV [3].

Despite these advances, HIV and its treatment pose significant clinical challenges in the context of advancing age [4]. PLWHs experience chronic, systemic inflammation, coined “inflammaging,” leading to accelerated aging and elevated risk for multiple age-related comorbidities [4,5], among which the most prevalent are cardiovascular disease, chronic kidney disease, cancer, metabolic disorders, and mental health and cognitive disorders, requiring complex care that extends beyond HIV management. Along with these factors, PLWHs have historically been a medically underserved population, facing stigma and negative social determinants of health (SDoHs), resulting in restricted access to healthcare and poor health outcomes [6]. The potential burdens associated with aging thus fall more heavily on PAWHs and require informed approaches to engage them as partners in their own healthcare. These factors highlight the need for healthcare strategies focusing on screening, prevention, and education in older PLWHs to mitigate HIV-associated health risks.

Patients’ perceptions, or mental models, of aging and health predict future health and well-being [7] and may be key to facilitate clinician–patient communication and shared decision-making. However, little is known about PAWHs’ perceptions of aging [8]. A general literature search found little/no qualitative data in the US on perceptions of aging among PAWHs, depriving clinicians of the guidance needed to address the evolving person-centered needs in this growing population. In the absence of patient testimony, clinicians often extrapolate patients’ needs from studies conducted outside the US [9,10,11,12] or from quantitative health measures [13,14], which paint an incomplete picture of the experience of living and aging with HIV [15,16,17,18] as predominantly negative [19,20]. HIV has commonly been equated with poor health, perpetuating a “medicalized view” that assumes well-being is inversely related to disease and medication burden [11,21,22,23].

A patient-centered approach is fundamental to the development of effective clinical interventions and preventive strategies, and qualitative research can provide nuanced insights into how HIV and related morbidities affect individuals as they age to elucidate their specific needs [13]. Drawing on the perspectives of PAWHs, this qualitative study explored how HIV and healthcare experiences influence perceptions of aging and health. These findings may inform healthcare providers of the needs of a population typically deemed underserved.

## 2. Materials and Methods

Design: This was a qualitative study consisting of one-time, semi-structured interviews with a convenience sample of PAWHs. Clinical details were verified through the EHR and linked directly to each participants’ unique ID code without any other identifying information. This study received IRB and institutional approval with waivers of need for written informed and HIPAA consent as all data were collected and recorded anonymously and signed consent forms would be the only identifying link to participation. All participants completed the informed consent process and gave verbal consent to participate.

Population and Setting: PAWHs aged ≥50 years and attending routine appointments over a 3-month period at an infectious disease specialty clinic associated with a large academic medical center and health system in a major metropolitan area were invited to participate. The integrated health system is the largest non-profit provider and largest private employer in New York state. It serves the greater New York metropolitan area, covering one of the most diverse sociodemographic populations in the US, and offers the full spectrum of services (i.e., from quaternary acute care to community hospital; full range of ambulatory primary and specialty care; subacute rehabilitation and skilled nursing care; and community-based care, including home care, emergency medical services, and hospice). The clinic specializes in acute and longitudinal HIV care and prevention in addition to general infectious disease services. It receives Ryan White Parts A and B funding.

Procedure: Patients checking in for appointments June–August 2024 on days when the study team was available were invited to participate. The treating physician introduced them to the study team and excused themselves during the consent and interview processes so as to be unaware if they decided against participating. Participants were informed that interviews would be captured in writing, omitting any personal identifying information. Interested participants underwent verbal informed consent and were allowed an accompanying support person to be present. Care team members were available for emotional support should the questions cause any distress. Interviews lasted 30–60 min and took place in a private examination room.

Interviews: The semi-structured interview guide included 14 open-ended questions that explored participants’ experience of aging and how HIV had impacted other health conditions, lifestyle behaviors, life priorities, and general age-related concerns (Appendix A). Potential participants were introduced to the interviewers after their appointments by their treating physician, who then left the room so as not to influence their decision to participate. The research team member explained the study, answered questions, and determined if the person was willing to participate. Participants were instructed to answer questions as briefly or fully as they wished and to stop at any time. Interviewers did not define “health” for participants, allowing them to respond based on their own understanding. Answers reflect each person’s unique interpretation and experiences.

All interviews were conducted by two authors (S.B. and M.R.). At the time of the interviews, S.B. was a post-baccalaureate student with experience as a research assistant and patient interviewer. M.R. was a second-year medical student. Both are advocates for the LGBTQ community. One conducted the interview and facilitated discussion, while the second transcribed the conversation, and they alternated roles between sessions. The transcriber would stop the conversation to verify quotes with participants as necessary, and both team members reviewed transcripts after completion of each interview to ensure the conversation was documented as accurately as possible. No audio recordings were made to encourage full and authentic responses while maintaining confidentiality. Interview transcriptions resulted in a mix of verbatim and gisted notes [24] which were typed directly into a password-protected file in a HIPAA-compliant database stored securely behind the health system firewall.

Transcripts had already been reviewed multiple times by the two interviewers when the first author began “actively reading” [25] to identify and note themes. Data were manually coded using an inductive approach following established principles of qualitative data analysis [24,25]. Data were grouped into overarching themes, linked to one another, and examined for redundancy and specificity. The entire final dataset was re-read, reviewed, and discussed by all authors to ensure themes were coherent. To enhance validity and credibility, all authors offered feedback until consensus was reached. Recruitment was concluded once thematic saturation was reached. The data were reviewed again in full by the first author, following the same coding process as described above.

## 3. Results

A total of 25 PAWHs were approached, and all agreed to participate and completed interviews. The average age was 63 years, with 13 men and 12 women who were racially, ethnically, and socioeconomically diverse; all the women and 38.5% of the men were heterosexual, 68.5% were men who had sex with men (MSM), and all were cisgender (Table 1). The duration of time living with HIV averaged 22.3 years, and 24 of 25 participants were virally suppressed. Two individuals reported receiving a first diagnosis of AIDS but both subsequently achieved viral suppression with ART and have maintained that status. Time living with HIV for these 2 individuals was 17 and 30+ years, respectively. First diagnosis for the remainder was “HIV-positive”, and none had a history of AIDS. Six individuals (one of those reporting having AIDS) were diagnosed before availability of highly active ART (HAART). All participants were assessed and documented as adherent to ART by the clinic pharmacist. Average time under care at the clinic was 19.4 years, and most participants had received continuous care at the same clinic since their initial HIV diagnosis (Table 1).

Participants appreciated the opportunity to recount their histories, expressing gratitude that HIV is no longer “a death sentence.” Most did not perceive any association between HIV and other chronic conditions; they perceived HIV as separate, controlled, and chronologically remote. Two important themes emerged from the questions on aging health: acceptance and/or fatalism about the future and fear of future debility.

### 3.1. Theme 1: Acceptance, Disengagement, and/or Denial

An overarching theme of acceptance and/or disengagement or even denial emerged for over half of respondents from questions about what lies ahead for their health. Themes of fatalism and/or acceptance (e.g., statements of inevitablity and helplessness) were illustrated by quotes such as “I know I’m getting older. Whatcha going to do about it?”; “It is what it is”; or “What are you going to do? This is life, you just have to accept it and move on.” Disengagement and even denial was common across the entire group (e.g., male, female, all ages, heterosexual, and MSM) illustrated by comments like “HIV is the least of my problems” (from an 82 year old); “You tell me I have HIV? I don’t know that. I only know that because of coming here.” (from a 54 year old); “I don’t really think about it; I forget it” (from a 66 year old); or “I don’t think about it at all” (from a 62 year old). God was described as the only one to determine longevity. Optimism, overcoming stigma and fear of disclosure, and personal responsibility for health were prominent subthemes voiced by younger participants (aged 51–67) who had strong social support and family ties.

#### 3.1.1. Optimism as Therapeutic Strategy/Relationship with Trusted Provider

Optimism, cultivated through interactions with medical personnel, was consistently described by participants as an essential therapeutic strategy. Nearly all participants highlighted the pivotal role of their providers in helping them develop the optimism necessary for life-time survival. As one shared, “It was so important to meet [my doctor] because he gave me the optimism to fight this long.” Another voiced similar ideas, “[My doctor] put in a lot of effort to help me think about the virus differently. Changing my mindset made me feel like I could live.”

Continuous care with a supportive provider was viewed as crucial to maintaining optimism. Physicians provided consistent reinforcement that healthy survival was possible and that aging well with HIV was an achievable reality. One participant explained how becoming optimistic was a critical first step toward initiating treatment. Diagnosed in the late 1980s, this individual recounted his five-year journey from denial and anger to acceptance and optimism. Initially refusing to become a “pill person,” he distrusted both medicine and those who prescribed it. His distrust was compounded by the stigmatizing treatment he received surrounding his original diagnosis, which led to loss of his support system and recalcitrant depression. Providing consistent non-judgmental support over five years, his doctor patiently earned his trust, providing education on safety of available therapeutics and helping him become receptive to starting antiretroviral treatment. Twenty-four years later, he has not only achieved viral suppression but self-manages his physical and mental health, reporting peace in daily life and acceptance for what lies ahead.

Optimism combined with prior experience remained a critical tool for contingent planning for possible future eventualities. For example, when asked about the level of care desired if incapacitated, the prior individual planned to transfer to an assisted living facility because he does not know anyone who could care for him at home. He had witnessed his mother in a similar position years before; although wishing his own circumstances were different, he has come to terms with this reality, reporting “acceptance” and believing things will turn out “okay.”

#### 3.1.2. The Role of the Clinic as a Safe Healthcare “Home”

Participants stressed not just the importance of their physician, but the entire interprofessional clinic staff in shaping their healthcare experiences and overall optimism. They spoke about the empathy and sensitivity shown by all staff, who were attuned to their unique needs. One participant noted, “Everyone is really nice, and that makes it easy to come back so regularly.” Another affirmed, “Every time I leave the clinic, I feel better.”

#### 3.1.3. Overcoming Stigma and Fear of Disclosure

Participants reported that overcoming stigma and fear of disclosure were important steps in openly discussing their health and future concerns, and one that improved their relationships and their overall health. Many described the distress and isolation caused by keeping their diagnosis secret from family and friends. Encouragement from medical providers helped them “come out” about their status to confidants and benefit from additional emotional support. For example, one of the oldest participants recounted, “I don’t know how I got it. [I’ve] never been with any man. [I] never thought this would happen to me. [I] kept it a secret from my wife, my kids, for three years. [My doctor] said you have to let people in. They’re your family. If you can’t confide in them, who can you confide in? I told them and they accepted me.” The quote from this participant implied that he associated HIV as a condition limited only to MSM. Due to anticipated potential (and internalized) stigma, this inaccurate perception deprived him of the support and acceptance he eventually received from family.

Likewise, a much younger participant shared that after years of keeping her status secret, she found the courage to disclose her diagnosis to her family, “I felt like I had to keep it all inside. I felt like people would reject me. It took me years until I could muster up the courage to tell my family. It was really weighing on my mind, and I just couldn’t take it anymore. When I did, they just loved me. No one pushed me away. Now, everyone knows. It’s my health.”

For this participant, overcoming fear of disclosure strengthened her family relationships, which restored her sense of purpose and drove adoption of healthy behaviors—“I want to live! I can’t lay around and mope. I have to get up and do things… I want to live. I like life. I want to be here. I know I’m here only till the good Lord takes me, but I want to be here for my mother, you know, and my kids.” In this excerpt, the theme of optimism intersects with a theme of personal responsibility for health—outlined below.

#### 3.1.4. Personal Responsibility Helps Reframe HIV from a “Death Sentence” to a Manageable Chronic Condition

The same participant quoted above described the need to take personal responsibility for her health: “I’m faithful when it comes to my meds, go to my doctor appointments, I keep my home nice, I’m clean. I volunteer. I go to therapy. I don’t care if you think I’m crazy. I need a stranger to talk to—to get this stuff off my chest.” This sense of personal responsibility for health was echoed by others and was central to how participants reshaped the narrative around HIV, transforming it from a “death sentence” to a manageable, chronic condition that allowed successful aging—“People who take their medications survive.”

Successful management of HIV as a chronic condition establishes behaviors that may be applied to other conditions and support healthy aging. Our participants reported taking an active role in their health through medication adherence, lifestyle changes, and regular medical care. As one participant noted, “How you age is up to you. I like to keep my body clean, shower, shave, look presentable. I take my meds religiously, and with good results.” Another emphasized, “I’m no spring chicken anymore. I don’t want this thing to make me look old. I go to the gym, keep my heart rate up, go to the doctor, stay useful.” (“Spring chicken” is an American idiom describing a person who is older and no longer in their youth.)

Though adherence was seen as paramount to controlling their disease, many acknowledged that lifelong adherence felt overwhelming at times. Developing discipline around medication regimens was essential. Some described themselves as “religious” about their meds, embracing spiritual aphorisms to manage the burden of lifelong adherence. For example—“I take my life one day at a time. Take my meds, exercise, eat well, don’t sweat the small stuff.”

### 3.2. Theme 2: Fear of Future Debility

Almost half of subjects expressed themes of fear and/or anxiety for the future, specifically around continued isolation with advancing age, dementia, and/or worry over finances. This was expressed more often by older participants (range 61–82), who lacked close relationships and were likely to express feelings of inevitability about declining health with advancing age.

#### 3.2.1. Social Isolation

##### Celibacy, Stigma, and Changing Social Landscapes

A lasting driver of isolation for PAWHs was the barrier HIV poses to finding romantic partners. Many stated that they could not forgive themselves if they inadvertently exposed a partner, despite knowing this fear was not grounded in current evidence, where “undetectable = untransmissible” [26,27,28]. Some of these individuals intimated they had contracted HIV from unfaithful past partners, which led them to write off relationships all together. Others identified fear of rejection as the major barrier—so powerful it convinced one participant to conceal his status from his current partner for one year before mustering up enough courage to disclose it.

A concern for stigma and desire for privacy around HIV was also common in this group. These participants reported feelings of shame at the prospect of disclosing their status to new friends and potential mates. As a result, some chose to remain both celibate and “incognito” about their status in their social lives, “mourning” the inability to truly connect with people as they could not be fully open.

For these already isolated individuals, natural changes to one’s social life over time, such as evolving neighborhoods, loss of friends, and relocating family, compounded their concerns about continued isolation with age. One older participant lamented, “The neighborhood has changed. I’m not saying I want to go back [to ‘back then’,] but every year it’s a new person moving in. We used to have block parties. No one knows each other anymore.” He went on to say that most of his friends had passed, and his children now live out of state; moving closer to them would mean giving up his long-standing relationship with his trusted provider, his “rock,” a compromise he was not willing to make. While he has attempted to connect with others through a nearby senior center, a sense of alienation within his community has lingered.

##### Support Groups

An additional layer of isolation emerged from feeling out of place in existing HIV support groups, which participants perceived as being geared primarily toward gay men. One participant stopped attending after gay group members joked about how he contracted HIV, insinuating homosexual transmission. Among heterosexual men, similar experiences fueled negative perceptions of support groups. COVID-19 introduced added barriers by initially eliminating in-person events and has remained a barrier for those who continue to take COVID precautions.

#### 3.2.2. Survivor Guilt

Many participants touched at least briefly on feelings of survivor guilt, having witnessed family and friends succumb to AIDS during the height of the epidemic. One of the most striking accounts came from a participant who was asymptomatic when first diagnosed with HIV. This participant was tested in hopes of becoming an organ donor for her dying child but was found to be HIV-positive. Early HIV treatment effectively revealed her status by making her visibly ill. As a result, she experienced significant rejection and stigma, particularly from family. She suffered severe depression following her child’s death, exacerbated by her family’s abandonment during this time of profound loss. Angry at God for “taking” her child (rather than herself), she stopped attending church. Still grieving, she recounted long nights of despair and many years marked by depression, dysfunction, and isolation.

Over time, this participant found meaning through service in the foster care system. Although expressing gratitude for this experience, the loss of her child and strained family relationships have colored her thoughts on aging. She recently began caring for her dementing father, magnifying resentment against her extended family who have distanced themselves from him. Seeing her father’s decline has added to her anguish. Reflecting on what lies ahead as she ages, she firmly asserted that she does not wish to live beyond 75. The thought of growing old, becoming physically debilitated, and continuing to carry decades of unrelenting grief felt unbearable.

#### 3.2.3. Memory Loss

Two participants reported recent cognitive difficulties at the time of their interview, and all others reported at least some concern about developing dementia. Most did not personally know similarly aged HIV-positive individuals suffering from dementia, but many had family members who developed dementia, and fear of succumbing to a similar fate was front of mind. A few shared memories of caregiving for loved ones with dementia or providing professional care working in a nursing home. Direct caregiving experience was a significant source of anxiety for these participants. They worried that any forgetfulness they were experiencing might indicate early cognitive decline and were interested in learning what, if anything, could be done to prevent or reverse its onset.

#### 3.2.4. Affording Aging

About a third of participants reported serious concerns about affording healthcare as they aged—retirement and loss of income, cost of medications, and being financially unprepared to pay for other conditions (e.g., cancer and dementia). These participants were interested in clinic-sponsored financial planning.

It is suggestive to note that the only participant who did not achieve viral suppression was the only individual reliant on public assistance. This participant had been receiving care from the clinic for 12 years and was one of the few who reported an unsatisfactory relationship with a provider. Upon further discussion, this individual reported frequently struggling with adherence, saying, “I go through spurts where I don’t want to take meds anymore. I know I have to, but I don’t want to.” Describing themselves as “overburdened by health conditions,” this individual expressed feelings of hopelessness, stating, “Before I got this, I wished I could live forever. Now I don’t. I don’t want to get past my sixties. I don’t want to be a senior citizen and HIV positive.” This situation is a sad reminder of the challenges faced by many PLWHs and the importance of SDoHs and mental resilience for aging more successfully [29].

## 4. Discussion

The major themes uncovered in these interviews reveal a clinically relevant disconnect between the biomedical understanding of aging with HIV and the illness perceptions held by many of the participants. Most of this group of PAWHs did not perceive a direct relationship between HIV and aging or development of future chronic health conditions. HIV had become a chronic but controlled and managed condition independent of other health issues. This can be effectively understood through the lens of the Common Sense Model of Self-Regulation (CSM) [30]. The model posits that individuals develop subjective, ‘common sense’ representations of their illnesses, which profoundly influence their coping behaviors and engagement with health advice. For many of the PAWHs in the group, absence of acute somatic symptoms and sustained viral suppression appears to have significantly reshaped their illness models, particularly across the “Identity, Timeline, and Consequences” dimensions of the CSM. The identity of HIV had shifted from an active, (potentially) life-threatening disease to a chronologically remote and ‘controlled’ condition. This perceived control fostered an implicit, and at times explicit, belief in a ‘resolved’ or ‘remitted’ illness timeline, rather than a continuous, lifelong process requiring vigilance beyond medication adherence. This phenomenon, where the lack of overt symptoms is equated with disease remission or ‘cure,’ is a well-documented pattern in patient perceptions across various chronic conditions, such as asthma, hypertension, and heart disease, often leading to reduced adherence or underestimation of risk [31]. Themes of personal responsibility suggest that many participants used a strategy of habit formation (e.g., “I take my meds religiously) to successfully “bypass” such inaccurate perceptions [32].

In general, the group appeared unaware or underestimated the potential for HIV’s systemic impact on “early onset of aging” and higher risk of developing many chronic illnesses. Participants often attributed emerging health issues to ‘just getting old,’ demonstrating a disconnect in their illness models regarding HIV’s role as a contributing ‘cause’ for these subsequent health challenges. This overlooks the sustained chronic ‘inflammaging’ associated with HIV, even in virally suppressed individuals, which drives an accelerated aging process and heightened vulnerability to conditions like cardiovascular disease, chronic kidney disease, and cognitive disorders [4,5]. This gap in understanding (particularly of the ‘causes’ and ‘consequences’ dimensions of their HIV perceptions) may pose a barrier to proactive engagement in comprehensive preventative care for other age-related conditions.

The unique profile of the participants further contextualizes these perceptions. About a third of the group had lived with HIV for 30 years or more and were diagnosed prior to or in the early era of HAART [3]. Their initial experience of HIV may have involved severe illness (reported by 4 participants), making their current state of viral suppression and relative health feel like a profound reprieve or ‘miracle.’ This history could strongly reinforce the perception of having ‘overcome’ HIV, thus reducing its perceived ongoing threat. Considering the diverse socioeconomic and demographic backgrounds of PAWH, often accompanied by histories of stigma and other social determinants of health, these personal illness models may also be shaped by varying levels of health literacy; trust in the healthcare system; and access to comprehensive, nuanced health information [6].

The strong and long-standing relationships with their healthcare providers and clinic team, as verbalized by our participants, while fostering optimism and adherence, may have inadvertently contributed to a reliance on feeling ‘well’ and being ‘taken care of,’ without fully internalizing the nuanced long-term risks. However, the comments suggest that these same strategies were strengths in maintaining health and function with aging. Many in the group were highly engaged in their care and well-versed in behaviors that promote healthy aging, despite having faced significant adversity. Many rated themselves as “healthier” than the general population. These successful strategies will be informative for the design and testing of future educational interventions and best communication strategies to improve engagement and health outcomes for PAWHs.

The themes that emerged from these interviews accord with previous findings of associations between social determinants of health and health experiences [29]. Older, less socially connected, and/or financially disadvantaged PAWHs were more likely to report experiences of anxiety, depression, prolonged stress, and a lack of agency over their aging experience. Because many PAWHs have limited support networks and resources, early educational interventions and assistance with financial planning may fill these gaps and improve outcomes for health and quality of life with aging, enabling PAWHs to maximize their options. Planning ahead for all PLWHs may ensure more equitable and efficacious care as they age.

Limitations: This study has a number of limitations that should be considered when interpreting the findings. There may have been selection bias in the sample as participants were introduced to the research team by their primary providers, with whom most had long-standing and trusting relationships. However, the fact that the informed consent process and interviews were conducted privately by two empathetic individuals who were not figures of authority suggests that the content of the interviews truly reflects the experiences and feelings of each individual. Although the sample size resulted in theme saturation and had nearly equal gender representation, additional themes might emerge if larger numbers of individuals from ethnic and racial minorities were included in the sample [30]. There is significant homogeneity in several other important areas: all participants were recruited from a single clinic located in a suburban area which may introduce site-specific biases related to clinical practices and patient demographics. They receive care within a large health system with a broad spectrum of resources compared to many other settings. The findings may not be applicable to patients located in urban or rural settings where care and patient demographics may differ. Finally, participants overall had a strong and long-standing connection with their healthcare providers/clinic and fewer unaddressed barriers to accessing care, which could influence their perceptions of aging and health. They have a higher than national average rate of viral suppression, which is likely related to this connection and may influence their perceptions of health.

## 5. Conclusions

This qualitative inquiry underscores that PAWHs face the complex reality of managing HIV alongside an accelerated burden of age-related chronic conditions, yet their internal illness perceptions often do not fully reflect this biomedical truth. This study highlights a crucial disconnect: while ART has dramatically improved survival, enabling PAWHs to age, many still view their HIV as an ‘overcome’ or ‘separate’ entity once virally suppressed. This common sense model of HIV may present a significant barrier to comprehensive care.

Healthcare providers may bridge this gap by developing and leveraging strong, trusting relationships (identified as central by our participants) to address and refine PAWHs’ existing illness representations. This involves explicitly clarifying that viral suppression is not a cure, and educating on the underlying mechanisms (e.g., inflammaging) and direct links between HIV and chronic comorbidities such as cardiovascular disease, renal decline, and neurocognitive impairment. By fostering a more accurate and nuanced understanding of HIV’s long-term ‘consequences’ and ‘timeline’ within patient-centered discussions, clinicians can better engage PAWHs in shared decision-making around preventative screenings, proactive management of comorbidities, and advanced care planning [21,22]. Tailored educational interventions, sensitive to the diverse experiences and social determinants of health influencing PAWHs, are vital to empower this vulnerable and growing population to maximize their health and quality of life as they age.

## Figures and Tables

**Table 1:** Demographics and clinical characteristics of participants (N = 25).

Age (mean ± SD/range)	63.5 ± 1.6/51–82
Female N (%)	12 (48)
Sexual Orientation	
Heterosexual (Male%, Female%)	68.50 (38.5, 100)
MSM (% of all Male)	61.5
Race/Ethnicity (N):	
Asian American	2
Black	12
Other?	1
White	10
Hispanic	4
Years Living with HIV (Mean ± SD/Range)	22.3 ± 9.6/1–38
Virally Suppressed N (%)	24 (96)
Years with Clinic (Mean ± SD/Range)	19.4 ± 8.1/1–30

MSM, men who have sex with men.

## Data Availability

The datasets presented in this article are not readily available because of the qualitative nature of the information and the need to maximize privacy and confidentiality. Requests to access anonymous transcriptions should be directed to Edith Burns, eburns4@northwell.edu.

## Abbreviations

The following abbreviations are used in this manuscript:

AIDS	Acquired immunodeficiency syndrome
HIV	Human immunodeficiency virus
MSM	Men who have sex with men
PAWHs	People aging with HIV
PLWHs	People living with HIV

## Appendix A. Interview Guide for Aging with HIV

1. Have you thought much about growing older and has that changed since finding out you were living with HIV? Do you feel differently now about living with HIV than when you first tested positive?
2. Have you noticed any changes in your body that you attribute to living with HIV?
3. Have you made changes in daily activities/schedule to make life easier? Have you changed plans you really wanted to do but felt you couldn’t because of your health?
4. Do you think about what is ahead for your health? How do you think you will feel about your health 10 years from now? Have your treatment goals changed over time?
5. Are there specific things you do to help your health?
6. What does your community look like? Do you have someone(s) to rely on in an emergency?
7. Tell me about how living with HIV has impacted your social life (if at all).
8. Do you have a healthcare proxy? (Yes or No) Do you have someone you trust to help make medical decisions if needed? Financial decisions? To help manage at home (e.g., cooking, cleaning, taking medication, home maintenance, dressing, bathing)?
9. Have you considered the level of care you would want if you had an incapacitating illness?
10. Do you know anyone living with HIV who developed memory problems? Do you have any family/friends who have struggled with dementia?
11. Have you had any concerns about memory loss as you get older?
12. How would you rate your health (excellent–very good–good–fair–poor)?
13. How would you rate your health compared to others of your age (excellent–very good–good–fair–poor)?
14. What additional services/resources from our office, social workers, nurses, and/or doctors would be helpful?

## References

[1] Blanco J.R., Jarrín I., Vallejo M., Berenguer J., Solera C., Rubio R., Pulido F., Asensi V., del Amo J., Moreno S. (2012). Definition of Advanced Age in HIV Infection: Looking for an Age Cut-off. AIDS Res. Hum. Retroviruses.

[2] Budack J.Z. (2023). HIV in Older Adults. University of Washington HIV Training Education Center. https://www.hiv.uw.edu/go/key-populations/hiv-older-patients/core-concept/all.

[3] Marcus J.L., Chao C.R., Leyden W.A., Xu L., Quesenberry C.P., Klein D.B., Towner W.J., Horberg M.A., Silverberg M.J. (2016). Narrowing the Gap in Life Expectancy Between HIV-Infected and HIV-Uninfected Individuals with Access to Care. J. Acquir. Immune Defic. Syndr. (JAIDS).

[4] Webel A., Schexnayder J., Cioe P., Zuñiga J. (2021). A Review of Chronic Comorbidities in Adults Living with HIV: State of the Science. J. Assoc. Nurses AIDS Care.

[5] Hart B., Nordell A., Okulicz J., Palfreeman A., Horban A., Kedem E., Neuhaus J., Jacobs D., Duprez D., Neaton J. (2018). INSIGHT SMART and ESPRIT Groups. Inflammation-Related Morbidity and Mortality Among HIV-Positive Adults: How Extensive Is It?. J. Acquir. Immune Defic. Syndr..

[6] Mahajan A.P., Sayles J.N., Patel V.A., Remien R.H., Ortiz D., Szekeres G., Coates T.J. (2008). Stigma in the HIV/AIDS epidemic: A review of the literature and recommendations for the way forward. AIDS.

[7] Benyamini Y., Burns E. (2019). Views on aging: Older adults’ self-perceptions of age and of health. Eur. J. Ageing.

[8] Carlsson Lalloo E., Acuña Mora M., Sundler A.J. (2025). Patient reported experiences in older adults living with HIV—A scoping review. AIDS Care.

[9] Lund M., Sundler A., Carlsson Lalloo E. (2024). Healthcare Experiences and Needs of Older Adults Living with HIV: A Qualitative Study. J. Clin. Nurs..

[10] Sharma A., Mwamba C., St Clair-Sullivan N., Chihota B., Pry J., Bolton-Moore C., Vinikoor M., Muula G., Daultrey H., Gittelsohn J. (2024). The Social Construction of Aging Among a Clinic-Based Population and Their Healthcare Workers in Zambia. Int. J. Public Health.

[11] Licchelli S., King A., Smith K. (2023). “It’s Still in the Test Tube and Finding out How the Experiment Ends…”. A Qualitative Study on Health and Aging in Older Gay Males Living with HIV in England. J. Int. Assoc. Provid. AIDS Care.

[12] Reynolds Z., Gilbert R., Sentongo R., Meyer A., Saylor D., Okello S., Nakasujja N., Greene M., Seeley J., Tsai A. (2022). Priorities for health and wellbeing for older people with and without HIV in Uganda: A qualitative methods study. J. Int. AIDS Soc..

[13] Rosenfeld D., Anderson J., Catalan J., Delpech V., Ridge D. (2021). How older people living with HIV narrate their quality of life: Tensions with quantitative approaches to quality-of-life research. SSM Qual. Res. Health.

[14] Byrd K.K., Buchacz K., Crim S., Beer L., Lu J., Dasgupta S. (2024). Unmet Needs for HIV Ancillary Services Among Persons with Diagnosed HIV Aged 55 years and Older. J. Acquir. Immune Defic. Syndr..

[15] Zhabokritsky A., Klein M., Loutfy M., Guaraldi G., Andany N., Guillemi S., Falutz J., Arbess G., Tan D., Walmsley S. (2024). Non-AIDS-defining comorbidities impact health related quality of life among older adults living with HIV. Front. Med..

[16] Drewes J., Ebert J., Langer P., Kleiber D., Gusy B. (2020). Social inequalities in health-related quality of life among people aging with HIV/AIDS: The role of comorbidities and disease severity. Qual. Life Res..

[17] Catalan J., Tuffrey V., Ridge D., Rosenfeld D., HALL Team (2017). What influences quality of life in older people living with HIV?. AIDS Res. Ther..

[18] Miners A., Phillips A., Kreif N., Rodger A., Speakman A., Fisher M., Anderson J., Collins S., Hart G., Sherr L. (2014). Health-related quality-of-life of people with HIV in the era of combination antiretroviral treatment: A cross-sectional comparison with the general population. Lancet HIV.

[19] Hsieh E., Polo R., Qian H., Fuster-RuizdeApodaca M., Del Amo J. (2022). Intersectionality of stigmas and health-related quality of life in people ageing with HIV in China, Europe, and Latin America. Lancet Healthy Longev..

[20] Emlet C.A. (2006). “You’re awfully old to have this disease”: Experiences of stigma and ageism in adults 50 years and older living with HIV/AIDS. Gerontologist.

[21] Pantelic M., Steinert J., Ayala G., Sprague L., Chang J., Thomas R., Nininahazwe C., Caswell G., Bach-Mortensen A., Bourne A. (2022). Addressing epistemic injustice in HIV research: A call for reporting guidelines on meaningful community engagement. J. Int. AIDS Soc..

[22] Fricker M. (2007). Epistemic Injustice: Power and the Ethics of Knowing.

[23] Biehl J., Eskerod T. (2007). Will to Live: Aids Therapies and the Politics of Survival.

[24] Lester J., Cho Y., Lochmiller C. (2020). Learning to Do Qualitative Data Analysis: A Starting Point. Hum. Resour. Dev. Rev..

[25] Braun V., Clarke V. (2006). Using Thematic Analysis in Psychology. Qual. Res. Psychol..

[26] Rodger A., Cambiano V., Bruun T., Vernazza P., Collins S., Degen O., Lundgren J. (2019). Risk of HIV transmission through condomless sex in serodifferent gay couples with the HIV-positive partner taking suppressive antiretroviral therapy (PARTNER2): A prospective observational study. Lancet.

[27] Grulich A., Bavinton B., Holt M., Prestage G., Zablotska I., de Wit J., Kaldor J. (2017). HIV transmission in serodiscordant male gay couples in Australia (Opposites Attract): An observational study. Lancet HIV.

[28] Rodger A., Cambiano V., Bruun T., Vernazza P., Collins S., Degen O., Lundgren J. (2016). Sexual Activity Without Condoms and the Risk of HIV Transmission in Serodifferent Couples When the HIV-Positive Partner Is Using Suppressive Antiretroviral Therapy. JAMA.

[29] Vrtikapa K., Hoque U., Hoque F. (2025). Social Determinants of Health: The Impact of This Overlooked Vital Sign. J. Brown Hosp. Med..

[30] Leventhal H., Brissette I., Leventhal E.A., Suls J., Wallston K.A. (2003). The common-sense model of self-regulation of health and illness. Social Psychological Foundations of Health and Illness.

[31] Leventhal H., Diefenbach M.A., Leventhal E.A. (1992). Illness cognition: Using common sense to understand treatment adherence and affect in chronic illness. Cogn. Ther. Res..

[32] Phillips L.A., Cohen J., Burns E., Abrams J., Renninger S. (2016). Self-Management of Chronic Illness: The Role of ‘Habit’ vs. Reflective Factors in Exercise and Medication Adherence. J. Behav. Med..

